# Root Canal Transportation after Root Canal Preparation with ProTaper Next, WaveOne Gold, and Twisted Files

**DOI:** 10.3390/jcm9113661

**Published:** 2020-11-14

**Authors:** Wojciech Eliasz, Kinga Kubiak, Wojciech Poncyljusz, Anna Surdacka

**Affiliations:** 1 Department of Conservative Dentistry and Endodontics, Poznan University of Medical Sciences, 60-812 Poznan, Poland; annasurd@ump.edu.pl; 2 Department of Diagnostic Imaging and Interventional Radiology, Pomeranian Medical University, 71-252 Szczecin, Poland; kinga_kubiak@yahoo.com (K.K.); wojciech.poncyljusz@pum.edu.pl (W.P.)

**Keywords:** endodontics, endodontic instruments, root canal preparation

## Abstract

Background: Root canal preparation during endodontic treatment may be associated with various complications, including a change in the original pathway of the root canal lumen. The aim of our study was to determine whether files of similar sizes that use various movement kinematics (rotary, reciprocal, adaptive motion) cause root canal transportation, and whether the differences between such systems are statistically significant. Methods: The degree of root canal transportation (DT) was calculated with the use of computed tomography scans for 3 groups of teeth (for each group: *n* = 20) in which the root canals were prepared using either rotary (ProTaper Next—PTN), reciprocal (WaveOne Gold—WOG), or adaptive movement (Twisted Files—TF) instruments. Results: For rotary ProTaper Next instruments, the mean value of the DT index was 0.0795 (SD = 0.0179) for 3 mm from the apex, 0.09 (SD = 0.0262) for 6 mm from the apex, and 0.106 (SD = 0.0221) for 9 mm from the apex. For reciprocal WaveOne Gold Primary instruments, the mean value of the DT index was 0.0355 (SD = 0.015) for 3 mm from the apex, 0.061 (SD = 0.02) for 6 mm from the apex, and 0.08 (SD = 0.25) for 9 mm from the apex. For Twisted Files, the mean value of the DT index was 0.05 (SD = 0.03) for 3 mm from the apex, 0.092 (SD = 0.17) for 6 mm from the apex, and 0.08 (SD = 0.02) for 9 mm from the apex. Conclusions: The use of PTN, WOG, and TF files resulted in root canal transportation to a different degree. The use of rotary PTN files produced the most transported preparation, whereas the use of WOG files produced the conservative root canal preparation that allowed the retention of the original shape of the root canal.

## 1. Introduction

Root canal preparation during endodontic treatment may be associated with various complications, including instrument separation, tooth fracture, a flare-up, and, e.g., change in the original pathway of the root canal lumen. As uneven removal of tooth structure, particularly radicular dentin, both decreases the resistance of the tooth to fracture and may eventually cause a perforation, one of the aims in manufacturing root canal instruments is to develop a tool that would cut the root canal dentin in the most uniform way possible [1]. Moreover, the aim of root canal preparation is not only to remove irreversibly damaged organic tissue, but also to shape the root canal in such a way that irrigating liquids and obturation materials can reach the appropriate levels [2]. That is why the so-called “non-instrumental” root canal preparation methods have not gained popularity. Even though endodontic files have different shapes, they all cut dentine in a manner that enlarges the root canal space in a conical shape, and the base of the cone is then circular. However, anatomical research studies have shown beyond doubt that root canals are, in the majority of cases, irregular-shaped, and are oval or ovoid in cross-section [3]. Eventually, this discrepancy causes uneven root canal preparation and only partial removal of debris due to the fact that more dentin is removed from one side of the canal. In the most extreme cases, such complications as, e.g., zip perforation (straightening of the root canal due to excessive removal of the external wall of curvature), elbow perforation (leaving a very thin wall of dentin next to the area of zip perforation), ledge formation, or direct perforation may occur, which will make the preparation of the root canal impossible. In the long-term perspective, such lesions may negatively influence the strength of the tooth, and, therefore, lead to fracture and treatment failure [4]. Therefore, several improvements in endodontic technology have been suggested in order to overcome the issue and minimize the risk associated with excessive removal of dentin in areas that are responsible for the resistance of the tissues. According to most research studies, this danger is encountered mostly when stainless steel instruments are used for preparation, as they do not conform to the original pathway of the root canal but rather “spring back” and retain their original shape. This is observed particularly when less experienced operators (e.g., students) perform the procedure. The use of nickel-titanium (NiTi) instruments may diminish this risk; however, there are several systems of NiTi instruments available for purchase, and they differ in various aspects—metallurgy, shape, and movement kinematics. Each of the aspects may influence their cutting efficacy and shape, and the outcome of their use has been studied extensively [5,6,7,8]. The aim of our study was to determine whether files of similar sizes that use various movement kinematics (rotary, reciprocal, adaptive motion) cause root canal transportation, and whether the differences between such systems are statistically significant.

## 2. Experimental Section

Sixty freshly extracted, single-rooted teeth with 1 root canal were chosen for the study (27 mandibular premolars, 27 maxillary premolars, 6 lateral maxillary incisors). The teeth were extracted due to universally agreed indications for extraction (non-restorability, periodontal disease, orthodontic indications). Inclusion criteria included: age (only teeth from patients aged 20–40 were chosen for the study), possibility of atraumatic extraction, absence of symptoms of parafunctions. Exclusion criteria included: root caries and pathological resorption. Only teeth with curvature up to 25 degrees were chosen. The teeth were divided into 3 groups (for each group *n* = 20), so that a tooth from each anatomical group would be included in each subgroup, and were then randomly classified into each of the subgroups using the Random Sequence Generator (Random.org, Dublin, Ireland) software. The methodology used for the study was adopted from the investigation by Elnaghy et al. [9]

The first stage of the experiment included a pre-operative CT scan. All scans were performed using the cone-beam computed tomography (CBCT) scanner Carestream CS 9300 (Carestream, Rochester NY, USA), with the following settings: small field of view (FOV) 5 × 5 cm, voxel size: 0.09 mm, scan time: 20 s. The teeth were placed in a silicon matrix, and lines denoting mesial, distal, buccal and palatal/lingual surfaces were marked. Afterwards, working length of the root canal was established. After access was prepared, an stainless-steel (SS) K-file (ISO 10, 2%) (Poldent, Warsaw, Poland) was placed in the root canal until its tip was visible at the apex, which was confirmed using a dental operating microscope—Leica M320 (Leica, Wetzlar, Germany). One millimeter was subtracted from this length, in order to set the working length (WL) at the physiological constriction. Afterwards, glidepath was prepared manually up to size 20/2% using manual SS K-files size 15/2% and 20/2% (Poldent, Warsaw, Poland). Then, the canals were prepared with the instruments, according to the manufacturers’ instructions:

- Group 1: ProTaper Next (PTN)—X-Smart endomotor (Dentsply Sirona, Charlotte NC, USA), 300 rpm, torque 2.0 N cm; size X1 and X2.
- Group 2: WaveOne Gold (WOG)—X-Smart endomotor (Denstply Sirona, Charlotte NC, USA)—WOG reciprocating mode; size: WOG Primary.
- Group 3: TF Adaptive (TF)—Elements Motor endomotor (Kerr Endodontics, Orange CA, USA)—Adaptive Motion program; size SM1 (20;0.04), SM2 (25;0.06), SM3 (35;0.04).

Between each instrument insertion, the root canal was rinsed with 1 mL of 0.9% NaCl.

In the next stage of the experiment, a post-operative CT scan was performed in the same matrix. The scans were then assessed using the Osirix MD (Pixmeo SARL, Bernex, Switzeland) software. The following values were measured:

- m1—the shortest distance from the mesial margin of the root to the mesial margin of the uninstrumented canal
- m2—the shortest distance from the mesial margin of the root to the mesial margin of the instrumented canal
- d1—the shortest distance from the distal margin of the root to the distal margin of the uninstrumented canal
- d2—the shortest distance from the distal margin of the root to the distal margin of the instrumented canal

The measurements were performed at the following levels: 3, 5, 7 mm from the apex. The degree of root canal transportation was calculated using the following equation:

(1) DT=(m1 − m2) − (d1 − d2)

**Figure 1:**
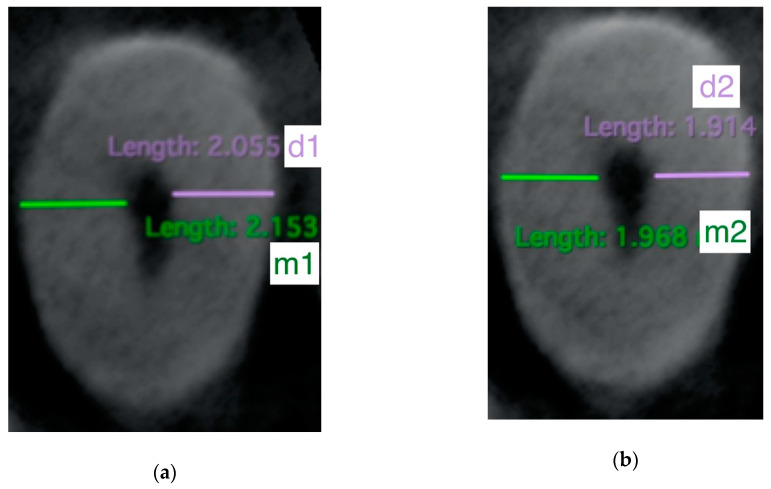
The cross-section of the root before (**a**) and after (**b**) preparation, m1 and m2 indicated in green, d1 and d2 in purple (m1—the shortest distance from the mesial margin of the root to the mesial margin of the uninstrumented canal, m2—the shortest distance from the mesial margin of the root to the mesial margin of the instrumented canal, d1—the shortest distance from the distal margin of the root to the distal margin of the uninstrumented canal, d2—the shortest distance from the distal margin of the root to the distal margin of the instrumented canal); measurements in mm.

An example of a root section with the measurements before and after preparation is shown in Figure 1. The calculations were made in the DICOM software.

Statistical significance level was set at α = 0.05. Data normality was checked using the Shapiro-Wilk test. For parametric data, multiple group ANOVA with Fisher’s post-test was used. For data that exhibited non-Gaussian distribution, Kruskal-Wallis ANOVA with Dunn’s post-test was used. The analyses were performed using the PQStat Software (PQStat, Poznan, Poland).

## 3. Results

For rotary ProTaper Next instruments, the mean value of the DT (degree of transportation) index was 0.0795 (SD = 0.0179) for 3 mm from the apex, 0.09 (SD = 0.0262) for 6 mm from the apex, and 0.106 (SD = 0.0221) for 9 mm from the apex.

For reciprocal WaveOne Gold Primary instruments, the mean value of the DT (degree of transportation) index was 0.0355 (SD = 0.015) for 3 mm from the apex, 0.061 (SD = 0.02) for 6 mm from the apex, and 0.08 (SD = 0.25) for 9 mm from the apex.

For Twisted Files that utilize adaptive motion, mean value of the DT (degree of transportation) index was 0.05 (SD = 0.03) for 3 mm from the apex, 0.092 (SD = 0.17) for 6 mm from the apex, and 0.08 (SD = 0.02) for 9 mm from the apex. The differences between each level for all systems are presented in Table 1.

Statistically significant differences for 3 mm from the apex were observed for Group 1 and Group 2 (*p* < 0.000001, Kruskal Wallis ANOVA), and Group 1 and Group 3 (*p* = 0.000459). For 6 mm from the apex, statistically significant differences were observed between Group 1 and Group 2 (*p* = 0.0004528, Kruskal-Wallis ANOVA), and Group 1 and Group 3 (*p* = 0.001254). For 9 mm from the apex, statistically significant differences were observed between Group 1 and Group 2 (*p* = 0.000763, ANOVA), and Group 1 and Group 3 (*p* = 0.014161, ANOVA). Mean differences in DT are presented in Table 2.

## 4. Discussion

Root canal instrumentation may be associated with several technical complications, including crack formation, apical extrusion of debris, and excessive change of the original root canal shape. Uneven dentin cutting during this process may weaken tooth structure, and thus, become a cause of treatment failure due to perforation, reinfection, and tooth fracture that renders the tooth non-restorable [10,11].

In order to determine the extent of this complication, the thickness of the root before and after preparation were compared using various imaging methods, such as CT [9], high resolution CT [12] or micro-CT [13]. Calculations drawn from this data were then used to determine how evenly the instruments remove dentin from the walls, and thus, these data can enable clinicians to reduce the risk associated with the endodontic procedure. The degree of root canal transportation, which was also used in our study, is one of the most commonly used and easy to interpret indices. This measure was used as it could be easily interpreted from the images acquired using a relatively widely available technology, i.e., CT. Calculations of other indices, e.g., the volume of removed tissue or other spatial measurements, are best performed with micro-CT imaging machines and may require editing in order to be realistic.

The results regarding the degree of root canal transportation (DT) in literature seem to indicate that the highest degree of DT is observed for instruments made of stainless steel, due to the metallurgical properties of such instruments. Files made of stainless steel undergo permanent deformation, and their stiffness causes them to force their way into the root canal. Therefore, with increasing sizes, they tend to make the root canal adapt to themselves, and to change its original path. Nickel-titanium instruments, on the other hand, adapt more easily to the shape of the canal, and can bend more easily. It is worth mentioning that root canal preparation with NiTi instruments is more predictable and more repeatable, even when performed by less experienced clinicians, e.g., students, at all stages of preparation, starting from the glidepath itself, which is viewed as difficult by many [14]. When it comes to glidepath preparation, there have been several novel systems introduced to the market, employing rotary and reciprocal motion, e.g., ProGlider (Dentsply Sirona, Charlotte NC, USA) or WaveOne Gold Glider (Dentsply Sirona, Charlotte NC, USA). Yet, there are no glidepath instruments that use adaptive motion. Moreover, manual preparation allows clinicians to detect any irregularities of the root canal and provide more feedback for them [15]. Therefore, in order to unify the pre-procedural steps, manual preparation was chosen. At this point it is worth mentioning that small engine-driven files have not always been shown to change the original pathway of the canal significantly [16].

Nevertheless, the variety of instrument systems available for purchase, and the differences in shape, metallurgy, enlargement protocols, and movement kinematics, account for the lack of uniformity and the difficulties in comparing them all. Furthermore, each of the aspects may influence the cutting properties of each instrument, and they all have been a subject of research studies worldwide. Our study aimed to determine whether the movement kinematics may influence the degree of root transportation, and, therefore, we used instruments of similar sizes that utilize different patterns of motion: rotary, reciprocal, and adaptive motion (rotary and reciprocal, depending on the conditions inside the root canal). Our results showed statistically different results between reciprocal and adaptive files, and rotary ones at each level of the root canal. Even though the issue is multifactorial, this may be attributed to the design of the file. The rotary files that were used in our investigation are rectangular in cross section but at the same time their center of rotation is offset. Therefore, as the distance from the tip of the instrument increases, the dentin is cut by the most externally positioned part of the file. For example, van der Vyver et al. also observed that ProTaper Next instruments remove more dentin when compared to other files [17]. This has been attributed to several factors, i.e., the so-called “envelope of motion”, thanks to which the final taper of the preparation may be greater than that of the original file. Even though this was theoretically disproven by Pasqualini et al. [18], the final taper of the preparation with PTN in their study was 6/7%, whereas the coronal part of all PTN files has 4% taper, which shows that the final shape of the preparation does not correspond to the shape of the file. Moreover, the off-centered shape of the instrument seems to play an important role in rotational movement only, as in reciprocal mode, the instrument does not utilize the envelope of motion phenomenon. In addition, pecking movements are performed while using WOG, and brushing of the root canal walls is done while using PTN, which may further explain the differences [17]. However, differences between the same file groups (WOG and TF) were determined to be statistically significant (*p* < 0.0001) by, e.g., Gergi et al. [19]. In our study, the differences at 3 and 9 mm from the apex were not significant, which may be interesting due to the fact that these instruments employ different motion kinematics and have different sizes. However, it may be the adaptive component of the movement that may account for the similar ratios [20]. All these groups have not yet been compared in any other study at the same time. However, when rotary and reciprocal systems were used, no statistically significant differences were observed between them, e.g., this was the case for PTN and TF [21], PTN and BT Race [22]. Furthermore, research studies conducted on other, commercially available systems did not exhibit statistically significant differences [23,24,25]. An interesting point about the study is that data regarding the relationship between working length and root canal transportation remain scarce. Most conclusions can be drawn in an indirect manner, by, e.g., evaluating dentinal crack formation after root canal preparation with WL set short of the apical foramen or apical constriction, directly at the apical foramen, and over the apex. It can generally be concluded on the basis of other research studies that overpreparation results in the formation of a larger number of cracks, which can further be attributed to excessive dental removal and change in the original shape of the root canal [26]. This may constitute a direction for further investigations.

An interesting point about the outcomes of our study is that preparation with reciprocating WaveOne Gold instruments resulted in significantly lower levels of root canal transportation when compared to other systems. However, in the studies by Wenzhe et al. [27] and Zhao et al. [28] the results were different, only the research team used the previous version of the instrument—the conventional WaveOne instrument made of NiTi alloy that was not treated thermomechanically in the same way as the Gold file, which acquires a gold-colored coating during manufacture. They concluded that a reciprocating motion resulted in the highest degree of root canal transportation. On the other hand, in the studies performed with WaveOne Gold instruments, such as by Özyürek et al. [29] and van den Vyver et al. [17], the conclusions were similar to our observation, i.e., that WOG produces more centered and less transported preparation, and its use is also associated with removal of smaller amounts of dentine than other files. There are two differences between the conventional WaveOne, and the WaveOne Gold system—the shape of the instrument, and the physical properties of the metal alloy. During the manufacture process, WOG instruments are subjected to various thermal processes whose aim is not only to increase their resistance, but also to make them more elastic and much more easily adaptive to the shape of the root canal [30]. Several studies have shown that the new instruments exhibit much better performance, as it takes longer for them to break, they can be put under higher levels of stress, and can overcome curvatures and other shape irregularities in a more predictable way than other instruments [31,32,33,34]. Therefore, it may seem that not only movement kinematics, but also the properties of the file itself may play a role in the transportation degree of the root canal. All these concepts should be taken into account in future research studies regarding endodontic preparation, as the conclusions drawn in meta-analyses until now show only that engine-driven preparation is more conservative and can, therefore, be viewed as safer [35].

## 5. Conclusions

The use of PTN, WOG, and TF files resulted in root canal transportation to a different degree. The use of rotary PTN files produced the most transported preparation, whereas the use of WOG files produced the conservative root canal preparation that allowed the retention of the original shape of the root canal.

## Figures and Tables

**Table 1:** *p*-value for the differences in DT at 9 mm from the apex.

	3 mm vs. 6 mm	6 mm vs. 9 mm	3 mm vs. 9 mm
PTN	0.0105	0.016	0.0265 *
WOG	0.0255 *	0.019 *	0.0445 *
TF	0.042 *	0.012 *	0.030 *

* Statistically significant difference. (DT—degree of transportation, PTN—ProTaper Next, WOG—WaveOne Gold, TF—Twisted Files).

**Table 2:** Mean differences in DT at 3, 6, and 9 mm from the apex between different file systems.

	3 mm from the apex	6 mm from the apex	9 mm from the apex
WOG vs. PTN	0.044 *	0.029 *	0.026 *
TF vs. WOG	0.015	0.031	0
TF vs. PTN	0.030 *	0.002 *	0.026 *

* Statistically significant difference. (DT—degree of transportation, PTN—ProTaper Next, WOG—WaveOne Gold, TF—Twisted Files).
